# Optimization of Taxol Extraction Process Using Response Surface Methodology and Investigation of Temporal and Spatial Distribution of Taxol in *Taxus mairei*

**DOI:** 10.3390/molecules26185485

**Published:** 2021-09-09

**Authors:** Lingyu Li, Yiming Chen, Yingli Ma, Zhong Wang, Tao Wang, Yinfeng Xie

**Affiliations:** 1 Co-Innovation Center for Sustainable Forestry in Southern China, College of Biology and the Environment, Nanjing Forestry University, Nanjing 210037, China; li13505153082@sina.com (L.L.); yli_ma@163.com (Y.M.); 2 Jiangsu Key Laboratory for the Research and Utilization of Plant Resources, Institute of Botany, Jiangsu Province and Chinese Academy of Sciences (Nanjing Botanical Garden Mem. Sun Yat-Sen), Nanjing 210014, China; m15261871180@163.com (Y.C.); wangzhong@cnbg.net (Z.W.)

**Keywords:** *Taxus wallichiana* var. *mairei*, taxol, response surface methodology, temporal and spatial distribution

## Abstract

*Taxus mairei* is an important source for industrial extraction of taxol in China. However, the standard and steps of extraction are currently not uniform, which seriously affects the taxol yield. In the present study, the influence of four factors (methanol concentration, solid-liquid ratio, ultrasonic extraction temperature, and ultrasonic extraction time) on the taxol yield was successively explored in *T*. *mairei*. A response surface methodology (RSM) was used to optimize the extraction process based on the single-factor experiments above. The optimal conditions were as follows: methanol concentration was 90%, solid-liquid ratio was 1:15 (g/mL), ultrasonic extraction temperature was 40 °C and ultrasonic extraction time was 60 min. Moreover, the twigs and needles from *T. mairei* with different tree ages were treated by the optimum extraction process, which further revealed temporal and spatial distribution of taxol in the reproducible tissues. Interestingly, the taxol content was relatively higher in needles of *T.* ‘Jinxishan’ (a cultivar from *T. mairei* with yellow aril, FY), but was less in FY twigs. The accumulation of taxol in twigs and leaves of females (with red aril, FR) was significantly higher than that of males (M); however, the content showed a decreasing trend with the increasing tree ages. Therefore, it is suitable to increase the proportion of female trees especially the FY leaves as raw materials for the industrial production of taxol from *T. mairei*, and the tree ages should be better controlled at 3–7 years.

## 1. Introduction

Taxol, a tetracyclic diterpenoid compound originally isolated from *Taxus brevifolia* [1], is considered one of the most effective anticancer drugs in the clinical treatment of leukemia, Kaposi’s sarcoma, ovarian, breast, and non-small cell lung cancers [2,3]. As the incidence of cancer increases, so does the commercial value of taxol [4]. However, the slow growth and extremely low taxol content of *Taxus* species lead to a fact that naturally extracted taxol is far from meeting the growing market demand [5]. Several alternative methods have been explored to increase taxol production in recent years, such as chemical total synthesis [6], in vitro *Taxus* cell culture [7], taxol-producing endophytic fungi [8], and heterogeneous synthesis [9], but most of them are difficult to commercialize due to their high cost and low production rate [10]. Therefore, the industrial extraction of taxol will continue to depend directly or indirectly on *Taxus* resources for the foreseeable future, while an efficient and sustainable method should be explored for the maximum acquirement of taxol, including the utilization of the reproducible tissues on *Taxus* and improvement of the extraction efficiency, which is necessary for the healthy development of taxol industry and the protection of *Taxus* from the endangerment.

Response surface methodology (RSM) is a statistical method for exploring optimal process parameters and solving multivariable problems by analysis of the response surface contours [11]. This method uses multiple quadratic regression equations to fit the functional relationship between the factors and the response values, which can not only visually show the influence of each factor on the yield, but also further analyze the interaction between various factors [12,13]. Recently, RSM has been widely used in optimizing the extraction process of active ingredients from different medicinal plants. Zou et al. [14] reported an ultrasound-assisted extraction technology of anthocyanins from mulberry based on RSM optimization, which promoted the yield to reach 64.70 ± 0.45 mg/g. The extraction of glycyrrhizic acid (GA) from licorice was optimized by Jiang et al. [15] using RSM, who reported the factors that significantly affect the extraction rate of GA and indicated that the interaction between the extraction time and the methanol concentration was crucial for GA extraction. RSM provides highly accurate predictions for the extraction of plant active ingredients, which therefore could be considered an appropriate strategy for the bulk extraction of taxol from *Taxus* trees.

*Taxus wallichiana* var. *mairei*, which belongs to *Taxus* genus in Taxaceae, is an evergreen tree with extremely high ornamental and material values [16,17]. Moreover, *T*. *mairei* is the fastest growing and most widely distributed tree species among *Taxus* plants with stronger adaptability to the external environment, so as to be the main source for industrial extraction of taxol in China [18,19]. Due to multiple and complicated extraction steps as well as inconsistent extraction standards, the taxol yield from *T*. *mairei* was seriously affected. In this study, RSM was used to optimize extraction process of taxol based on a series of single-factor experiments. Moreover, the twigs and leaves from different *T*. *mairei* trees with different tree ages were treated by the optimum extraction conditions to investigate the temporal and spatial distribution of taxol in *T*. *mairei*. The results provide scientific reference for the improvement of taxol extraction technology as well as the rational development and utilization of *Taxus* resources.

## 2. Materials and Methods

### 2.1. Plant Material and Growth Conditions

The male (M) and female (with red aril, FR) trees of *T. mairei* and individuals of *T.* ‘Jinxishan’ (a cultivar selected from *T. mairei* with yellow aril, FY) were chosen as experimental materials and were planted in the fields of Nanjing botanical garden Mem. Sun Yat-Sen, Nanjing China (32°3′ N, 118°49′ E). This area belongs to the subtropical humid climate zone, with an annual average temperature of 16.2 °C and an annual average precipitation of 1013 mm. The soil used in this field was yellow-brown soil. The soil was neutral (pH = 6.68), mixed with suitable organic fertilizer, containing 6.79 g organic matter, 1.86 g nitrogen, 127 mg available phosphorus, 295 mg available potassium per kg soil, and less than 1 mg mercury per kg soil. The plants per cultivar at the same ages were cultivated closely and under same management conditions. In this study, the samples of leaves in 7-year-old FR were collected in mid-March 2019 for optimization of the extraction process of taxol. Moreover, the twigs and leaves from 7-year-old M, FR, and FY trees, and those from 3- and 15-year-old FR trees were additionally collected in mid-March 2020 to investigate the temporal and spatial distribution of taxol in *T*. *mairei*. Each sample was collected from 3 plants. The collected samples were washed and dried at 60 °C for about 6 h, and then were powdered for determination of taxol content.

### 2.2. Taxol Extraction

The powder (0.2 g) from M, FR, and FY was accurately weighed and transferred into a 10 mL centrifuge tube with methanol solution. Ultrasonic extraction was carried out using an ultrasonic apparatus (KQ5200DE, Kunshan Ultrasound Instrument Company, Kunshan, China) with a frequency of 40 kHz and fixed power dissipation of 200 W under the set conditions of methanol concentration, solid-liquid ratio, ultrasonic extraction temperature, and time (details were shown in the design of single-factor experiments). The sample was then centrifuged at 10,000 rpm for 7 min to collect the supernatant. After drying by nitrogen blowing instrument, the dried residue was dissolved in 1 mL of methanol solution. Then, the solution was filtered by 0.22 μm microfiltration membrane for the determination of taxol content.

### 2.3. Taxol Content Determination

The determination of taxol content was carried out using Agilent 1100 high-performance liquid chromatography (HPLC) and an Agilent diode array detector (DAD). The separation of taxol was achieved on a penomenex Curosil-PFP column (250 mm × 4.6 mm, 5 μm). The mobile phase was composed of acetonitrile and water through the following gradient eluted program: 0–35 min, 30:70 (*v*/*v*); 35–60 min, 50:50 (*v*/*v*); 61–70 min, 30:70 (*v*/*v*). The detection wavelength was set to 227 nm with the flow rate of 2.6 mL/min under 30 °C, and an injection volume of 20 µL. A standard solution of taxol was employed to create a standard curve for quantification. The standard taxol (≥99.9 %, CAS: 33069–62-4) used for reference purpose was purchased from the National institutes for food and drug control (Beijing, China). The standard curve for analysis of taxol was obtained using the following equation (Equation (1)):

Y = 2012.1X + 12.6 (R^2^ = 0.9982) (1)

where Y was the peak area and X was the concentration of standard taxol with the linear range of 5.0–20 μg/mL.

### 2.4. Taxol Content Calculation

The taxol content was calculated using the following formula (Equation (2)):


Taxol content (μg/g) = (C × V)/M
(2)

In the formula above, C means the concentration of taxol obtained from the standard curve (μg/mL), V means the total volume after constant volume (mL), and M means the *T. mairei* samples powder quantity (g).

### 2.5. Single-Factor Experiment Design

The influence of methanol concentration (60%, 70%, 80%, 90% and 100%), solid-liquid ratio (1:5, 1:10, 1:15, 1:20, and 1:25 g/mL), ultrasonic extraction temperature (20 °C, 30 °C, 40 °C, 50 °C, and 60 °C) and ultrasonic extraction time (10 min, 20 min, 40 min, 60 min, and 90 min) on the taxol yield was studied, respectively. The fixed levels of four factors were set in turn to 90%, 1:15 g/mL, 40 °C, and 60 min. When a factor was studied, other factors were set as fixed levels.

### 2.6. Response Surface Experiment Design

In order to obtain the optimal process for the extraction of taxol from *T. mairei*, the experiments were performed on the Box–Behnken Design (BBD) according to the results of a single-factor experiment. The methanol concentration (A), solid-liquid ratio (B), extraction temperature (C), and extraction time (D) were chosen as the independent variables. The taxol yield was selected as the responses for the combination of the independent variables. According to previous research, the low, medium, and high levels of each independent variable were coded with −1, 0, 1, respectively. Experimental factors and levels are shown in Table 1.

### 2.7. Statistical Analysis

All the experiments were carried out in triplicate, and the results were expressed as means ± SE of three biological replicates. The experimental results of the response surface design were analyzed using Design-Expert 8.0.6 software. The plots were produced using Design-Expert 8.0.6 and Origin 8.5 software. Statistical analysis was conducted with SPSS 19.0 software using one-way analysis of variance (ANOVA) and Duncan multiple comparisons. A value of *p* < 0.05 was considered statistically significant, *p* < 0.01 was considered statistically highly significant and *p* < 0.001 was considered statistically extremely significant.

## 3. Results

### 3.1. The Influence of Four Factors on Yield of Taxol

#### 3.1.1. Effect of Methanol Concentration on Yield of Taxol

**Figure 1:**
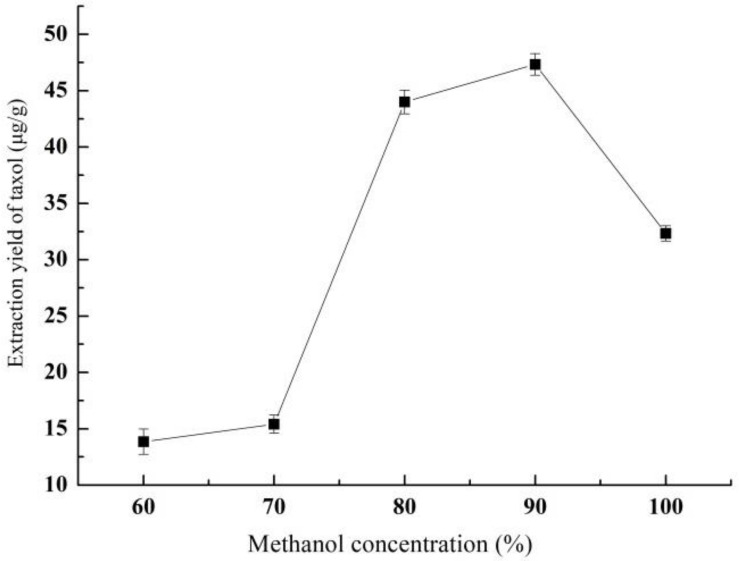
The effect of methanol concentration on the yield of taxol.

Figure 1 shows the effect of methanol concentration on the taxol yield. With the increase of methanol concentration, the taxol yield showed a trend of first increasing and then decreasing. The taxol yield increased rapidly from 15.41 ± 0.80 μg/g to 44 ± 1.05 μg/g as the methanol concentration increased from 70% to 80%, and finally the maxima (47.33 ± 0.96 μg/g) appeared at 90% of methanol concentration.

#### 3.1.2. Effect of Solid-Liquid Ratio on Yield of Taxol

**Figure 2:**
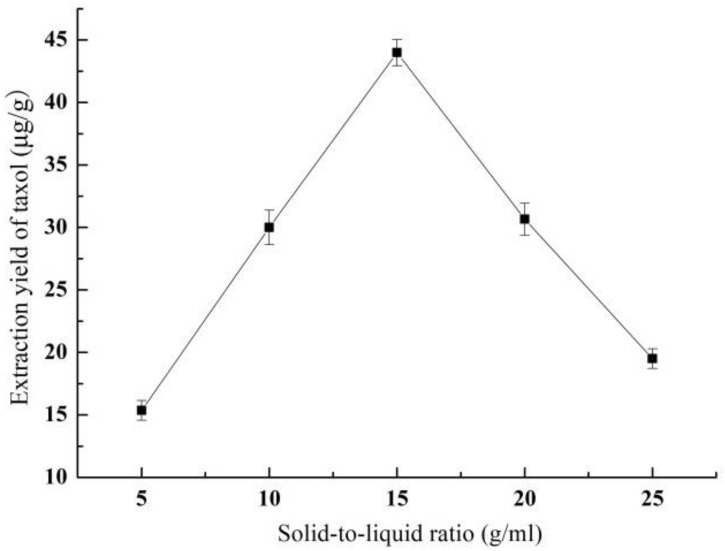
The effect of solid-liquid ratio on the yield of taxol.

Figure 2 shows the effect of the solid-liquid ratio on the taxol yield. The taxol yield increased rapidly with the increase of the solid-liquid ratio until the peak value (43.99 ± 1.04 μg/g) appeared at the solid-liquid ratio of 1:15 (g/mL). However, as the solid-liquid ratio continued to rise, the taxol yield decreased gradually. The increase of solid-liquid ratio promotes the dissolution of taxol by reducing the mass transfer resistance; but a large amount of solvents also promotes the dissolution of inert components and competitive solutes, such as waxes and pigments, which results in a smaller taxol yield. Therefore, the optimal solid-liquid ratio was 1:15 (g/mL).

#### 3.1.3. Effect of Extraction Temperature on Yield of Taxol

**Figure 3:**
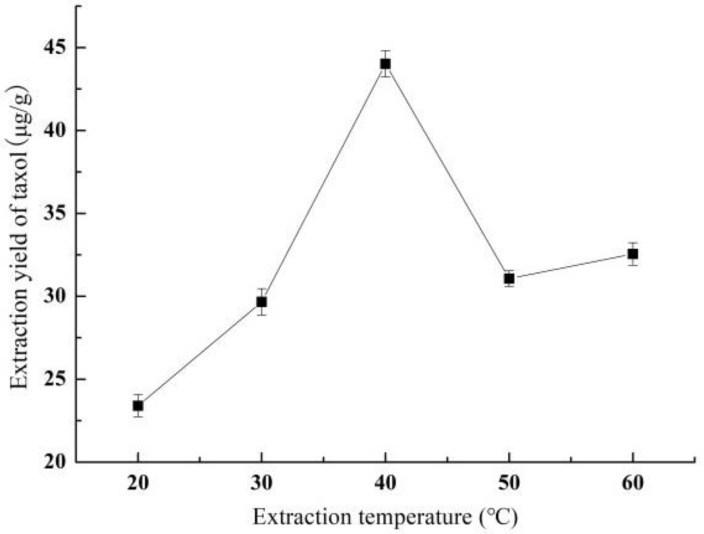
The effect of extraction temperature on the yield of taxol.

Figure 3 shows the effect of extraction temperature on the taxol yield. The taxol yield increased rapidly from 23.40 ± 0.67 to 44.02 ± 0.80 μg/g with the increase of the extraction temperature from 20 °C to 40 °C. Subsequently, the taxol yield showed a trend of decrease at first and then gradually flattening as the extraction temperature continued to increase. The rise in temperature facilitates taxol diffusion by decreasing viscosity; but the higher temperature also causes degradation of taxol. Thus, the optimal extraction temperature was 40 °C.

#### 3.1.4. Effect of Extraction Time on Yield of Taxol

**Figure 4:**
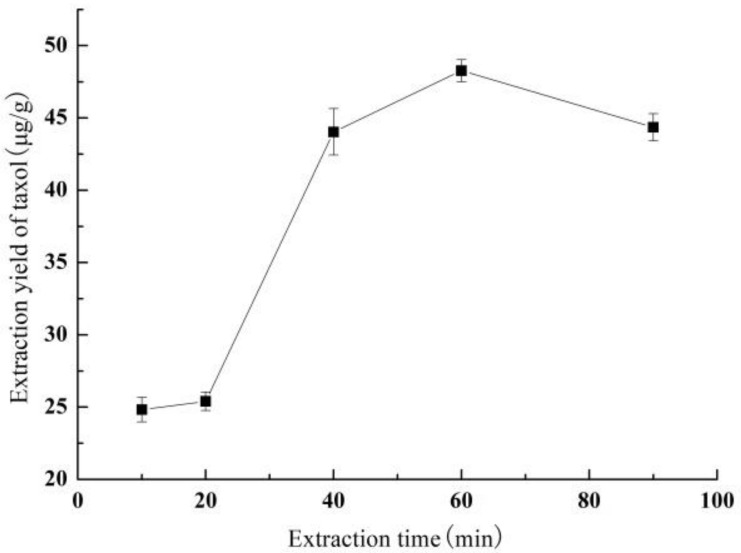
The effect of extraction time on the yield of taxol.

Figure 4 shows the effect of extraction time on the taxol yield. The taxol yield showed a trend of first increasing and then decreasing with the increase of extraction time, which was similar to that of methanol concentration. Interestingly, the taxol yield increased rapidly from 25.40 ± 0.64 μg/g to 44.04 ± 1.60 μg/g as the extraction time increased from 20 min to 40 min, and finally the maxima (48.27 ± 0.76 μg/g) appeared at 60 min of extraction time. With the extraction time continuing to increase, the taxol yield showed a slow downward trend.

### 3.2. Optimization of the Extraction by RSM

The taxol yield of *T. mairei* was further optimized through the RSM approach. The coded and actual levels of the four variables in Table 1 were selected to maximize the yield. Twenty-nine experiments were designated, in which 24 were factorial experiments and 5 were zero-point tests performed to estimate the errors. Table 2 shows the treatments with coded levels and the experimental results of taxol yield. The yield ranged from 36.27 to 50.01 μg/g. The maximum yield was recorded under the experimental conditions of A = 90%, B = 1:15 (g/mL), C = 40 °C, and D = 60 min. The data from BBD were analyzed by multiple regression to fit the following quadratic polynomial model (Equation (3)):

Y = 49.97 − 2.78A + 0.58B + 0.67C + 0.25D − 0.082AB + 0.49AC + 0.082AD − 0.26BC + 0.095BD − 0.073CD − 3.79A^2^ − 4.97B^2^ − 5.95C^2^ − 2.35D^2^ (3)

where Y is the yield of taxol (μg/g) and A, B, C, and D were the coded variables for methanol concentration, solid-liquid ratio, extraction temperature, and extraction time, respectively.

To illustrate the validity of the regression equation and the influence of each factor on the extraction rate, ANOVA was conducted on the above regression model. As shown in Table 3, the *p*-value of the regression model was <0.001, indicating that the model was extremely significant. The lack of fit was used to verify the adequacy of the model. The lack of fit *p*-value was >0.05, and the determination coefficient (R^2^) was >0.99. These combined results indicated that the model could adequately fit the experiment data, and was suitable to predict the yield of taxol of *T. mairei*. Moreover, the linear term and quadratic term were extremely significant (*p* < 0.001). This indicated that the selected factors significantly affect the taxol yield. According to the F-Value, the order of the influence of each factor on the taxol yield was methanol concentration (A) > extraction temperature (C) > solid-liquid ratio (B) > extraction time (D).

**Figure 5:**
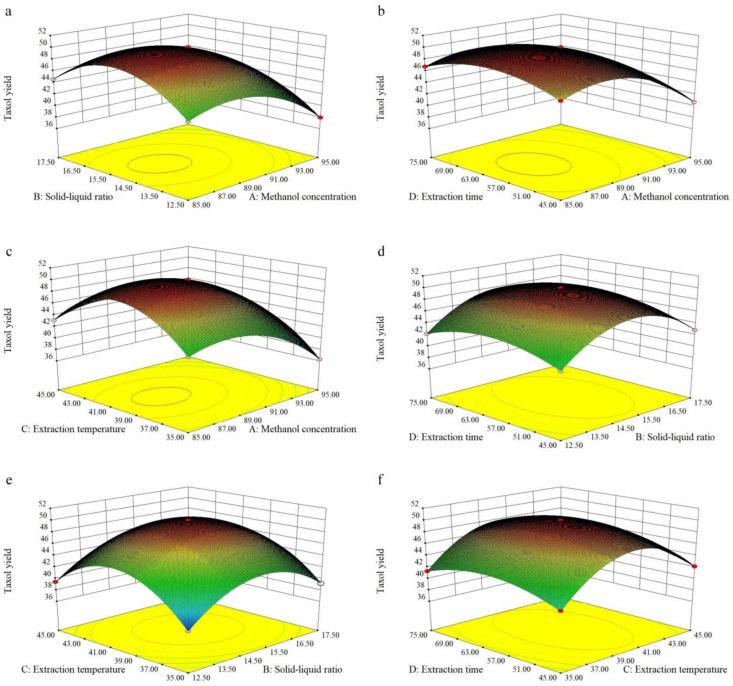
Response surface plots of the effect of factor interactions on taxol yield. (**a**) Effects of methanol concentration and solid-liquid ratio on the yield of taxol; (**b**) Effects of methanol concentration and extraction time on the yield of taxol; (**c**) Effects of methanol concentration and extraction temperature on the yield of taxol; (**d**) Effects of solid-liquid ratio and extraction time on the yield of taxol; (**e**) Effects of solid-liquid ratio and extraction temperature on the yield of taxol; (**f**) Effects of extraction temperature and extraction time on the yield of taxol.

**Figure 6:**
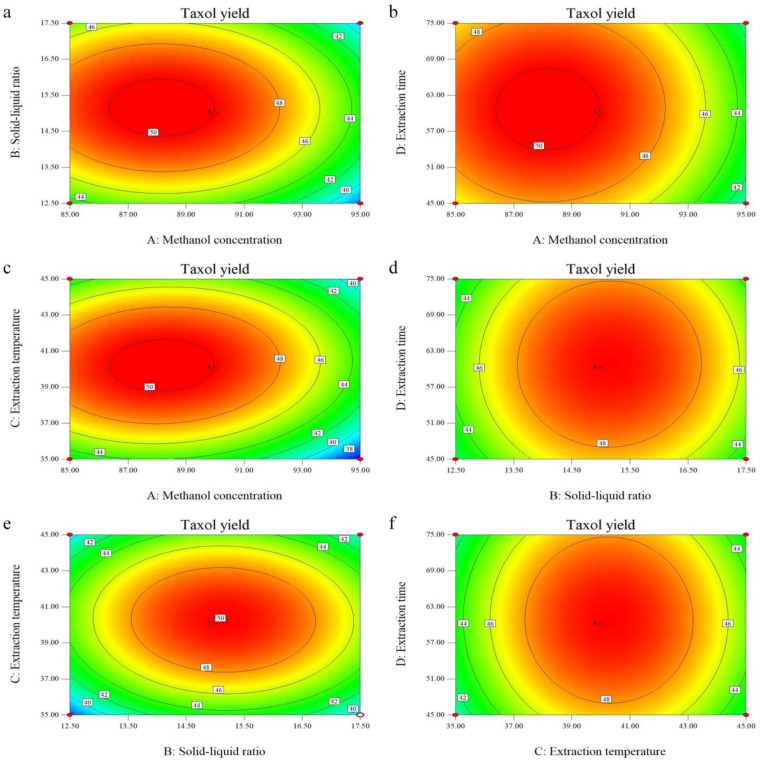
Contour plots of the effect of factor interactions on taxol yield. (**a**) Effects of methanol concentration and solid-liquid ratio on the yield of taxol; (**b**) Effects of methanol concentration and extraction time on the yield of taxol; (**c**) Effects of methanol concentration and extraction temperature on the yield of taxol; (**d**) Effects of solid-liquid ratio and extraction time on the yield of taxol; (**e**) Effects of solid-liquid ratio and extraction temperature on the yield of taxol; (**f**) Effects of extraction temperature and extraction time on the yield of taxol.

The three-dimensional (3D) response surface plot and 2D contour plot are the graphical representations of the regression equation, and the results of the taxol yield, consisting of the methanol concentration (A), solid-liquid ratio (B) extraction temperature (C), and extraction time (D), illustrate the pairwise interactions between the factors [15]. In the present study, all the surfaces were upper convex with a maximum value at the center of the response surface, due to the negative quadratic coefficients in the regression equation, which also approves the rationality of the predicted models [20]. It can be seen from the response surface and corresponding contour lines in Figure 5 and Figure 6, respectively, that the slope of factor AC, AB, and BC was relatively steep and its contour was elliptical, indicating that the pairwise interaction of the three factors of A, B, and C was very significant; while the slope of factor AD, CD, and BD was slightly flat, and the contour was round, indicating that the extraction time had relatively weak influence on the response value. The above results are consistent with the results of the variance analysis.

After calculation by Design Expert software, the optimum extraction conditions were determined according to the response surface optimization as follows: methanol concentration was 88.17%, solid-liquid ratio was 1:15.15 (g/mL), extraction temperature was 40.20 °C, and extraction time was 60.73 min, the maximum yield of taxol was (50.01 ± 0.09) μg/g. We then combined the practical feasibility of the operation, made little adjustment to the above parameters and adjusted the extraction conditions of the methanol concentration to 90%, solid-liquid ratio to 1:15 (g/mL), extraction temperature to 40 °C, and extraction time to 60 min. Under these conditions, the actual value of taxol extracted in three parallel experiments was 49.97 ± 0.15 μg/g, which clearly showed that the model fitted the experimental data and was considered to be accurate and reliable for predicting the taxol yield.

### 3.3. Comparison of Taxol Content from Different T. mairei Cultivars

**Figure 7:**
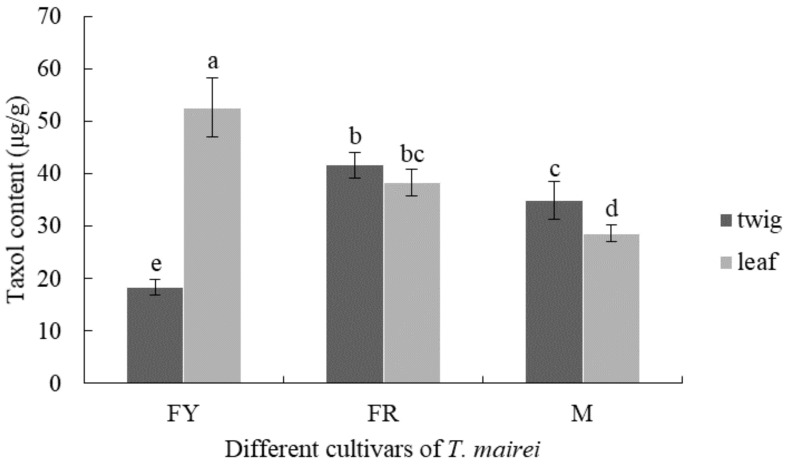
Taxol content in twigs and leaves among different *T. mairei* cultivars. Different letters in the same series indicate significant difference at *p* < 0.05 level.

Figure 7 shows the taxol content in twigs and leaves from 7-year-old FY, FR, and M, respectively. Among them, the highest taxol content was observed in FY leaves, which was up to 52.7 μg/g, while that in FY twigs was the lowest (18.3 μg/g), and the values were significantly different (*p* < 0.05). On the contrary, the taxol content in leaves of FR and M was lower than that in twigs, but significant difference was only observed in twigs and leaves of M (*p* < 0.05). Most notably, both leaves and twigs of FR showed a higher accumulation of taxol in comparison to those of M with significant difference, and the highest taxol content in FR was 41.7 μg/g.

### 3.4. Comparison of Taxol Content from T. mairei with Different Tree Ages

**Figure 8:**
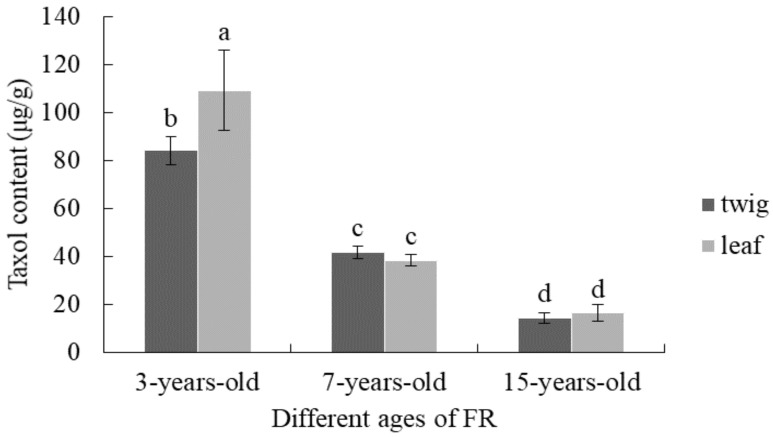
Taxol content in twigs and leaves of FR with different ages. Different letters in the same series indicate a significant difference at *p* < 0.05 level.

Figure 8 shows the taxol content in twigs and leaves of FR with different ages. Among them, the taxol content in twigs and leaves of 3-year-old FR was relatively higher, which was 84 μg/g and 109 μg/g, respectively, and the difference was significant (*p* < 0.05). Interestingly, the accumulation of taxol in both twigs and leaves showed a decreased trend with the increase of tree ages, but the difference of average taxol content between twigs and leaves in 7- and 15-year-old FR trees was not significant (*p* > 0.05).

## 4. Discussions

Taxol is one of the most effective antitumor drugs in the world and is approved by the U.S. Food and Drug Administration (FDA) for the treatment of various cancers [21]. However, the diverse and complex steps of industrial extraction of taxol, such as the selection of solvent and the methods of auxiliary extraction, caused significant differences in extraction efficiency and yield. In fact, solvents such as methanol, ethanol, acetone, dichloromethane, and trichloromethane have commonly been used for extraction of taxol from *Taxus* and the methanol was proved to be the best solvent for the extraction among these solvents [22], which was in accordance with the characteristics of taxanes soluble in alcohol solvent and has the advantages of wide application and low cost [23]. In the present study, methanol was chosen for the taxol extraction. Interestingly, the taxol yield showed a decreasing trend as the volume fraction of methanol exceeded 90%. This was due to the increasing volume fraction of methanol that could promote not only the dissolution of taxol, but also the dissolution of impurities that compete with taxol for solvent [24]. Moreover, a proper solid-liquid ratio was also crucial for the extraction as the higher or lower solid–liquid ratio led to inadequate extraction, which was disadvantageous for the ultrasonic assisted extraction [25]. Ultrasonic extraction technology could contribute to the release, diffusion, and dissolution of active substances in the cells. The enhancement of the mass transfer brought about by acoustic-induced cavitation in a liquid medium is one of the main advantages [26]. This mechanism increases the extraction efficiency of ultrasonic extraction by several times to dozens of times compared with ordinary reflux, dipping and decoction methods, and the energy consumption per unit material is more than 50% lower than that of conventional methods, which has been widely used in the extraction of active components from medicinal plants in recent years [27]. In this study, a proper solid-liquid ratio combined with ultrasonic extraction significantly increased the yield of taxol, but higher extraction temperature and longer time also showed opposite effects, which was related to the characteristics of taxol itself. Similar results were reported in other studies about the taxol extraction [28]. Therefore, the factors of methanol concentration, solid-liquid ratio, ultrasonic extraction temperature, and time were selected to optimize the extraction process of taxol in the present study, so as to maximize the taxol yield from *T. mairei*, which possessed excellent application values.

A series of statistical methods could be used to optimize the extraction process of taxol from *T. mairei*, for example the orthogonal design and RSM. The traditional orthogonal design mainly uses representative experiment cases instead of comprehensive cases for the process optimization, while the preferred values could only be a certain combination of selected experiment cases [29]. In contrast, RSM could make up for these shortcomings, and even provide additional information of interaction between various factors through regression equations and 3D graphics [30]. In this study, the extraction process of taxol from *T. mairei* was optimized using RSM based on four selected factors mentioned above, of which the order of the influence of each factor on the taxol yield was methanol concentration > extraction temperature > solid-liquid ratio > extraction time. Boyadzhieva et al. [31,32] reported that after determining the extraction solvent, study on the extraction temperature should precede the solid-liquid ratio, which induced high yield without assisting to thermal destruction of the active component. This was basically consistent with our results. Moreover, with the increase of extraction time from 20 min to 60 min, the taxol yield increased significantly. However, the results of RSM showed relatively weak pairwise interaction between extraction time and other factors and influence on the taxol yield. The possible reason was that RSM optimization was valid for the chosen interval close to the optimal values selected in the single factor experiment. According to the optimal model analysis, the ideal extraction conditions were eventually determined to be methanol concentration of 90%, solid-liquid ratio (g/mL) of 1:15, ultrasonic extraction temperature of 40 °C, and ultrasonic extraction time of 60 min. This improved method showed high repeatability, stability, and accuracy. Combined with the results of temporal and spatial distribution of taxol, this method could also be widely used in different cultivars of *T. mairei*, which provided new insight into the industrial extraction of taxol.

Taxol is one of most important and special secondary metabolites in *Taxus* trees. The slight difference of taxol contents in leaves of FR with same tree ages, which was determined mid-March 2019 and 2020, indicated that the accumulation of taxol was highly influenced by the external environments, which was consistent with the literature reported by Yu et al. [33]. Moreover, the taxol content is also vulnerable to several internal factors, including the tree genders, ages, and tissues [34]. In the present study, the reproducible tissues of *T. mairei* with different cultivars and ages were treated by the optimized methods reported above. The results showed that the taxol content in FY leaves was the highest, which indicated a promising source for industrial extraction of taxol as well as an excellent material for the research of taxol biosynthesis. The taxol content in the twigs and leaves of FR was significantly higher than that of M, implying that the synthesis and metabolism of taxol may be affected by gender differences. However, this was not exactly the same as the results reported by Nadeem et al. [35] and Fett-Neto et al. [36]. The reason could be attributed to the different *Taxus* germplasms or tissues. Moreover, with the increasing ages of *Taxus* trees, the taxol content in both twigs and leaves of FR showed a decreasing trend. Fang et al. [37] reported a phenomenon regarding metabolic transfer of taxol in *Taxus* tissues, which was considered a special survival mechanism for *Taxus* plants. This was similar to our results and could explain the situation in the present study. Therefore, the twigs and leaves from *T. mairei* of 3–7 years old were ideal for the extraction of taxol. Considering the advantages of female cultivars in extracting taxol as well as the relatively high content of taxol in the leaves of female trees with different ages, the leaves from female trees are more recommended to be used for obtaining significant quantities of taxol, which further enriched the understanding of selection of reproducible tissues in *T. mairei*.

## 5. Conclusions

On the basis of single-factor experiments, the optimal values in the factors of methanol concentration, solid-liquid ratio, ultrasonic extraction temperature, and time were determined, which showed significant effects on the taxol yield. The RSM was used to establish the regression equation model between the response values of taxol yield and these four factors. The conditions of optimal extraction process were obtained as follows: methanol concentration was 90%, solid-liquid ratio was 1:15 (g/mL), ultrasonic extraction temperature was 40 °C, and ultrasonic extraction time was 60 min. Moreover, the twigs and leaves of *T*. *mairei* with different cultivars and ages were treated by the optimal extraction process. Among them, the highest taxol content was observed in the leaves of FY, which indicated a promising source for industrial extraction of taxol in the future. The taxol content in both twigs and leaves of FR was significantly higher than that of M; however, the content showed a decreasing trend with the increasing ages of *Taxus* trees. The reason was mainly attributed to the gender differences and metabolic transfer of taxol within different *Taxus* tissues. In general, it is more recommended to use female tree leaves to obtain large amounts of taxol. This further enriched the understanding of selection of source in *T*. *mairei* for the industrial extraction of taxol.

## Figures and Tables

**Table 1:** Levels and factors of the response surface experiment.

Factor	Level		
	−1	0	1
Methanol concentration (%)	85	90	95
Solid-liquid ratio (g/L)	12.5	15	17.5
Extraction temperature (°C)	35	40	60
Extraction time (min)	45	60	75

**Table 2:** Response surface design and experimental data.

No.	Methanol Concentration (%)	Solid-Liquid Ratio (g/mL)	Extraction Temperature (°C)	Extraction Time (min)	Taxol Yield (μg/g)
1	90	15	35	45	40.72
2	85	15	40	75	46.81
3	90	15	40	60	49.99
4	90	12.5	45	60	39.46
5	85	17.5	40	60	44.65
6	95	15	35	60	36.27
7	90	15	40	60	49.94
8	90	12.5	35	60	37.52
9	95	15	40	75	41.38
10	85	12.5	40	60	43.32
11	90	15	40	60	50.01
12	90	12.5	40	75	42.21
13	90	15	40	60	49.93
14	95	15	40	45	40.71
15	90	15	45	75	42.51
16	90	15	35	75	41.34
17	85	15	45	60	43.16
18	95	17.5	40	60	38.96
19	85	15	35	60	42.82
20	90	17.5	35	60	39.18
21	90	17.5	45	60	40.08
22	90	17.5	40	45	42.85
23	90	17.5	40	75	43.58
24	90	12.5	40	45	41.86
25	90	15	45	45	42.18
26	85	15	40	45	46.47
27	90	15	40	60	49.98
28	95	15	45	60	38.56
29	95	12.5	40	60	37.96

**Table 3:** Test of significance for content of taxol from *T. mairei*.

Source	Degree of Freedom	Sum of Squares	Mean Square	F-Value	*p*-Value	Significance
model	14	463.52	33.11	17,960.09	<0.0001	***
A	1	92.91	92.91	50,398.74	<0.0001	***
B	1	4.05	4.05	2196.10	<0.0001	***
C	1	5.47	5.47	2965.90	<0.0001	***
D	1	0.77	0.77	417.77	<0.0001	***
AB	1	0.03	0.03	14.77	0.0018	**
AC	1	0.95	0.95	515.68	<0.0001	***
AD	1	0.03	0.03	14.77	0.0018	**
BC	1	0.27	0.27	146.68	<0.0001	***
BD	1	0.04	0.04	19.58	0.0006	***
CD	1	0.02	0.02	11.41	0.0045	**
A^2^	1	93.23	93.23	50,575.76	<0.0001	***
B^2^	1	160.30	160.30	86,957.85	<0.0001	***
C^2^	1	229.64	229.64	124,569.44	<0.0001	***
D^2^	1	35.75	35.75	19,390.49	<0.0001	***
Residual	14	0.03	0.00	-	-	-
Lack of fit	10	0.02	0.00	1.84	0.2913	-
Pure error	4	0.00	0.00	-	-	-
Total	28	463.55	-	-	-	-

*** represents the difference is extremely significant (*p* < 0.001). ** represents the difference is highly significant (*p* < 0.01).

## Data Availability

Not applicable.

## Sample Availability

Samples of the compounds are not available from the authors.
